# Oral and general health-related quality of life in patients treated for oral cancer compared to control group

**DOI:** 10.1186/s12955-014-0201-5

**Published:** 2015-01-23

**Authors:** Rocío Barrios, Manuel Bravo, Jose Antonio Gil-Montoya, Ildefonso Martínez-Lara, Blas García-Medina, Georgios Tsakos

**Affiliations:** Research Fellow of the Spanish Ministry of Education, School of Dentistry, University of Granada, c/Llanete del Mercado n 5, 23680 Alcalá la Real, Jaen Spain; Preventive and Community Dentistry, School of Dentistry, University of Granada, C/Campus Cartuja s/n, 18071 Granada, Spain; Special Care in Dentistry and Gerodontology, School of Dentistry, University of Granada, C/Campus Cartuja s/n, 18071 Granada, Spain; Oral and Maxillofacial Surgeon, Servicio de Cirugía Maxilofacial, Hospital Universitario “Virgen de las Nieves”, Avenida de las Fuerzas Armadas, 2, 18014 Granada, Spain; Department of Epidemiology and Public Health, Dental Public Health, Institute of Epidemiology and Health, University College London, 1-19 Torrington Place, London, WC1E6BT UK

**Keywords:** Oral cancer, Quality of life, SF-12, OHIP, OIDP

## Abstract

**Background:**

Health-related quality of life (HRQoL) is gaining importance as a valuable outcome measure in oral cancer area. The aim of this study was to assess the general and oral HRQoL of oral and oropharyngeal cancer patients 6 or more months after treatment and compare them with a population free from this disease.

**Methods:**

A cross-sectional study was carried out with patients treated for oral cancer at least 6 months post-treatment and a gender and age group matched control group. HRQoL was measured with the 12-Item Short Form Health Survey (SF-12); oral HRQoL (OHRQoL) was evaluated using the Oral Health Impact Profile (OHIP-14) and the Oral Impacts on Daily Performances (OIDP). Multivariable regression models assessed the association between the outcomes (SF-12, OHIP-14 and OIDP) and the exposure (patients versus controls), adjusting for sex, age, social class, functional tooth units and presence of illness.

**Results:**

For patients (n = 142) and controls (n = 142), 64.1% were males. The mean age was 65.2 (standard deviation (sd): 12.9) years in patients and 67.5 (sd: 13.7) years in controls. Patients had worse SF-12 Physical Component Summary scores than controls even in fully the adjusted model [β-coefficient = −0.11 (95% CI: −5.12-(−0.16)]. The differences in SF-12 Mental Component Summary were not statistically significant. Regarding OHRQoL patients had 11.63 (95% CI: 6.77-20.01) higher odds for the OHIP-14 and 21.26 (95% CI: 11.54-39.13) higher odds for OIDP of being in a worse category of OHRQoL compared to controls in the fully adjusted model.

**Conclusion:**

At least 6 months after treatment, oral cancer patients had worse OHRQoL, worse physical HRQoL and similar psychological HRQoL than the general population.

**Electronic supplementary material:**

The online version of this article (doi:10.1186/s12955-014-0201-5) contains supplementary material, which is available to authorized users.

## Background

Incidence rates have increased for oral cavity and oropharyngeal cancers in recent years [1]. Improvement in the treatments has resulted in a decrease in mortality [2] and consequently more patients than ever before are living with the sequelaes of the illness [3]. These sequelaes could affect their quality of life [4]. Thus, the measurement of health-related quality of life (HRQoL) is gaining importance as a valuable outcome measure, particularly in the oral cancer area.

Health-related quality of life is a concept that reflects a subjective measurement of health status, commonly assessed by generic or disease-specific questionnaires. Generic questionnaires provide valuable information by interpreting functional status in the broader scope of the patient’s life [5]. Moreover, as they are not specific for oral cancer, they potentially allow comparisons with populations free from this disease. However, due to the complex anatomy of the oral cavity it is desirable to complement generic HRQoL measures with the use of specific oral health-related quality of life (OHRQoL) measures. These questionnaires are more sensitive in assessing the impact of oral conditions on daily life [6].

A relevant question in oral cancer patients is to assess the degree to which patients adapt to the treatment effects and recover their habitual lifestyle post-treatment. Long-term HRQoL assessment including a comparison group would aid to answer this question and would improve the interpretations of findings [7]. Relevant studies in homogeneous samples of oral and oropharyngeal cancer patients have been inconclusive. Some found that patients had lower scores (worse HRQoL) [8,9] and others found similar scores or even higher scores (better HRQoL) [10-13] compared to reference values. Moreover, most studies compared the results with population norms and only one, focused on physiological problems, used a control group [11].

The aim of this study was to assess the general and oral HRQoL of oral and oropharyngeal cancer patients in Granada, Spain, 6 or more months after treatment and compare them with a population sample free from this disease.

## Methods

### Patients and controls

A sex and age group frequency matching study was conducted from January 2011 to January 2014. The study base was the population of Granada, a province in Southern Spain. All people diagnosed with oral cancer were referred to the *Virgen de las Nieves* University Hospital. Therefore, the patients of our study were selected from the Department of Maxillofacial Surgery of that hospital. Inclusion criteria for participation in the study were: patients treated for oral or oropharyngeal cancer, treatment has finalized at least six months before the recruitment to the study and the patients were free from recurrence of the disease. In such a study, the controls should come from the same population than the cases. Therefore, the controls were selected from different settings in Granada (social centers, geriatric centers and companions of hospital patients) with the following inclusion criteria: not diagnosed for oral cancer and belonging to one of the sample strata (sex and age group).

Cases and controls were grouped into sex and age group strata that were matched to have the same frequency. We only considered age and sex in the frequency matching to avoid an excessive numbers of strata in the sampling procedure which could make it impractical. Other relevant variables, such as sociodemographic factors, were instead considered in the statistical analysis as confounding factors and were adjusted for in multivariable associations. A total of 145 cases and 146 controls fulfilled the inclusion/exclusion criteria and were initially selected. Of them, 3 cases and 4 controls did not accept to participate in the study, giving 142 cases (97.9% acceptance rate) and 142 controls (97.3% acceptance rate) for the analysis.

Our sample sizes, 142 cases and 142 controls, is sufficient to detect, with a significance level α = 0.05 and power = 80% (β = 0.20), a standardized difference of 0.3 in the outcome between patients and controls, which is between small (0.2) and moderate (0.5) (according to Cohen’s scale [14]).

The study was approved by the Ethics Committee of the University of Granada and signed an informed consent was obtained from each participant.

HRQoL and OHRQoL measures were treated as the outcome variables, study group (patients or controls) as the main exposure and sex, age, social class, presence of illness (comorbidities) and functional tooth units as covariates. Functional posterior tooth units were defined as pairs of occluding natural, restored or fixed prosthetic postcanine teeth (molars = 2 units; bicuspids = 1 unit) [15]. Functional anterior tooth units were defined as pairs of occluding natural, restored or fixed prosthetic precanine teeth (each tooth = 1 unit). Moreover, specific data of the tumor and treatment (tumor location, clinical stage, date of treatment completion and type of treatment) was collected for the patients.

### Measurement of HRQoL

The 12-Item Short Form Health Survey was used to evaluate HRQoL. The 12-Item Short Form Health Survey (SF-12) is a reduced version of one the most commonly used general questionnaire, the 36-Item Short Form Health Survey (SF-36). The version 2 of SF-12 is a useful tool with the advantages of its brevity versus SF-36 and the possibility of calculating the 8 original dimensions versus the version 1 [16,17]. This validated instrument [18] contains 12 ítems with 3- or 5-point Likert scales. These items result in 8 dimensions: Physical Functioning, Role Physical, Bodily Pain, General Health, Vitality, Social Functioning, Role Emotional, and Mental Health. Two summary scores, Physical Component Summary and Mental Component Summary, are calculated from these dimensions.

The derivation of SF-12 scores followed established procedures [19]. First, we calculated the scores of the 8 dimensions and transformed them to a 1–100 scale; then, the scores were standardized and finally a lineal transformation was done. The lineal transformation was done taking the values 50 and 10 as sample estimate of the mean and standard deviation respectively of the reference general population. Computations of the aggregate summary components consist of multiplying each of eight standardized dimensions by its respective physical or mental factor score coefficient, and then summing the eight products. The last step also involves transforming the aggregate physical and mental summary scores to the norm-based (50, 10) scoring. We chose the specific method for the calculation using theSF-12 reference standards for the Spanish population [16]. Higher scores indicate better quality of life.

### Measurement of OHRQoL

OHRQoL was assessed through two widely used relevant generic measures: the Oral Health Impact Profile (OHIP-14) and the Oral Impacts on Daily Performances (OIDP). The Oral Health Impact Profile (OHIP-14) comprises 14 items that explore seven dimensions of impact: functional limitation, physical pain, psychological discomfort, physical disability, psychological disability, social disability and handicap. The participants respond to each item according to the frequency of the impact on a 5-point Likert scale (ranging from 0 to 4): never, hardly ever, occasionally, fairly often, and very often [20]. The additive score (OHIP-A) scoring method was used where the total score was calculated summing the item codes for the 14 items. The OHIP-14 extent was calculated as the number of individual items affected by impacts occasionally or more frequently.

The Oral Impacts on Daily Performances (OIDP) index assesses the impact of oral conditions on eight daily performances: eating, speaking, cleaning teeth, carrying out major work or role, social contact, relaxing/sleeping, smiling, and emotional state. It evaluates the frequency and the severity of these impacts through Likert scales. For each performance a score is calculated by multiplying the frequency and severity scores. The sum of these performances scores is divided by the maximum possible score and multiplied by 100 to give a percentage overall score. In addition, the OIDP extent was calculated as the number of performances affected [21,22].

For both the OHIP-14 and the OIDP, a higher score indicates worse OHRQoL. The recall period for both was changed from the usual 12 or 6 months to 1 month in patients and controls. As cases were interviewed at least 6 months after the end of their oral cancer treatment, we used a 1-month time reference in order to avoid including the acute period of recovery, in the cases of recent treatment.

### Statistical analysis

Statistical analysis was performed using the SPSS version 17.0 software package (SPSS Inc., Chicago, IL). Descriptive analysis of socio-demographic variables, SF-12, OHIP and OIDP was followed by bivariate associations between the covariates and study group (patients or controls) using the appropriate test according to the type of variable (chi squared for categorical variables, t-test for continuous normally distributed and Mann–Whitney for continuous skewed variables).

Linear regression models evaluated the differences in the summary components of SF-12 between patients and controls. Because the OHRQoL outcome variables were not normally distributed in our sample, we evaluated the unadjusted and adjusted associations of OHIP-14 and OIDP with the study group (patients versus controls) using ordinal multimodal regression models. The OHIP-14 extent and OIDP extent were categorized as the dependent variables (0 = 0 items affected; 1 = 1–2 items affected; 2 = 3–4 items affected and 3 = 5 or more items affected). The first adjusted model accounted for the effect of all socio-demographic variables (age, gender and social class). We sequentially added the only oral health variable that was significant in the bivariate model (posterior functional teeth) to construct the second adjusted model and the final model was built by additionally accounting for the effect of general health (presence of illness).

Possible differences in outcome variables (SF-12, OHIP and OIDP) with respect to the origin of controls (social centers versus geriatric centers versus companions of hospital patients) were evaluated with Kruskal-Wallis and ANOVA test.

Bivariate associations between clinical and treatment data of the patients were evaluated using the appropriate test according to the type of variable (t-test and ANOVA test for continuous normally distributed and Mann–Whitney test and Kruskal Wallis test for continuous skewed variables).

The level of statistical significant was set to *p* < 0.05.

Furthermore, to assess the clinical importance of the difference in HRQoL and OHRQoL between patients and controls we calculated the standardized effect size [23] for SF-12, OHIP-14 and OIDP.

Authors have followed the STROBE guidelines for carrying out the study and for writing the paper [24].

## Results

The descriptive data and bivariate associations between study group and socio-economic variables are shown in Table 1. Overall, 64.1% were males. The mean age was 65.2 (standard deviation (sd): 12.9) years in patients and 67.5 (sd: 13.7) years in controls. More than half of the patients and controls belonged in the lowest social class (V). No significant differences were found between these two groups with respect to the sociodemographic data or the presence of diseases. The patients had significantly fewer posterior functional tooth units compared to the controls.

**Table 1:** **Socio-economic and clinical data variables description of oral cancer survivors and controls**

**Variable**	**Patients**	**Controls**	***p***
	**n (%)**	**n (%)**	
All	142 (100)	142 (100)	
Sex			1.000^a^
Male	91 (64.1)	91 (64.1)	
Age (years)			
<50	18 (12.7)	18 (12.7)	
50-65	54 (38.0)	54 (38.0)	
>65	70 (49.3)	70 (49.3)	
range	29-90	26-93	
mean ± sd	65.2 ± 12.9	67.5 ± 13.7	0.151^b^
Social Class^d^			0.790^c^
I	8 (5.6)	8 (5.6)	
II	8 (5.6)	9 (6.3)	
III	14 (9.9)	8 (5.6)	
IV	35 (24.6)	45 (31.7)	
V	77 (54.2)	72 (50.7)	
Functional tooth units			
Anterior (mean ± sd)	2.4 ± 2.7	3.0 ± 2.7	0.072^b^
Posterior (mean ± sd)	2.4 ± 3.6	3.9 ± 4.5	0.004^b^
Presence of diseases^e^			0.886^c^
No	29 (20.4)	30 (21.1)	
1	57 (40.1)	53 (37.3)	
2 ó more	56 (39.5)	59 (41.6)	
Tumor site			
Tongue	50 (35.2)		
Buccal mucosa	18 (12.7)		
Mouth floor	16 (11.3)		
Gingiva	16 (11.3)		
Oropharynx	16 (11.3)		
Others	26 (18.3)		
Cancer stage			
I	61 (43.0)		
II	25 (17.6)		
III	17 (12.0)		
IV	39 (27.5)		
Follow-up (years)			
0.5-5	92 (64.8)		
6-10	33 (23.2)		
11-20	17 (12.0)		
Mean ± sd	4.9 ± 4.3		
Treatment			
S^f^	74 (52.1)		
S + RT^g^	43 (30.3)		
S + RT + CH^h^	25 (17.6)		

^a^chi-square test with Yates continuity correction; ^b^Student’s t test; ^c^Mann–Whitney test; ^d^In descending order; ^e^Chronic diseases; ^f^S: surgery; ^g^RT: radiotherapy; ^h^CH: chemotherapy.

The most frequent location for oral cancer was the tongue and the clinical stages I and IV were the more prevalent. The mean follow-up was 4.9 (sd: 4.3) years and the most common treatment was surgery without adjuvant radiotherapy and/or chemotherapy.

In relation to the SF-12, significant differences between patients and controls were found in the following dimensions: Role Physical, Bodily Pain and General Health. Patients had significant worse Physical component summary. These differences were not significant in Physical functioning, Vitality, Social Functioning, Role Emotional, Mental Health dimensions and in the Mental component summary (Table 2).

**Table 2:** **Comparison of health-related quality of life (SF-12) between oral cancer survivors (n = 142) and controls (n = 142)**

**Variable**	**Cases**	**Controls**	***p*** ^**a**^
	**Mean ± sd**	**Mean ± sd**	
Physical functioning	41.2 ± 13.0	42.7 ± 12.2	0.295
Role physical	40.7 ± 13.0	44.6 ± 12.0	0.009
Bodily pain	46.4 ± 9.2	50.0 ± 7.0	<0.001
General health	44.0 ± 6.2	45.9 ± 7.9	0.023
Vitality	45.4 ± 7.9	46.6 ± 8.6	0.227
Social functioning	43.0 ± 12.5	45.6 ± 9.6	0.055
Role emotional	44.7 ± 12.6	47.2 ± 10.0	0.063
Mental health	44.8 ± 8.9	45.1 ± 7.9	0.732
Physical component summary^b^	42.2 ± 12.0	45.5 ± 11.2	0.019
Mental component summary^c^	45.8 ± 11.6	46.6 ± 8.9	0.509

^a^Student’s t test; ^b^The effect size of the difference was 0.28 (95% CI: 0.05-0.51); ^c^The effect size of the difference was 0.08 (95% CI: −0.15-0.31).

In terms of the OHRQoL, there were statistically significant differences between patients and controls in all the domains or items and in the overall score of both questionnaires. The largest differences were in physical disability, physical pain and functional limitation in the OHIP-14 and in speaking, eating and emotional status in the OIDP. The domains/performances with highest score (worse impact) were similar for both groups (patients and controls); these referred to physical pain for the OHIP-14 and eating difficulty for the OIDP (Table 3).

**Table 3:** **Comparison of oral health-related quality of life (OHIP-14 and OIDP) between oral cancer survivors (n = 142) and controls (n = 142)**

**Variable**	**Cases**	**Controls**	***p*** ^**a**^
	**Mean ± sd**	**Mean ± sd**	
OHIP-14^b,c^			
Functional limitation	3.3 ± 2.2	1.4 ± 2.1	<0.001
Physical pain	3.9 ± 2.4	1.8 ± 1.9	<0.001
Psychologycal discomfort	2.7 ± 2.5	0.6 ± 1.2	<0.001
Physical disability	3.7 ± 2.9	1.5 ± 2.1	<0.001
Mental disability	1.6 ± 2.2	0.2 ± 0.9	<0.001
Social disability	1.6 ± 2.2	0.1 ± 0.5	<0.001
Handicap	2.1 ± 2.1	0.3 ± 0.7	<0.001
Overall	18.9 ± 11.8	5.9 ± 6.2	<0.001
OIDP^d,e^			
Eating	9.8 ± 7.2	3.9 ± 4.8	<0.001
Speaking	7.8 ± 6.5	0.7 ± 1.8	<0.001
Cleaning teeth	1.1 ± 3.3	0.1 ± 0.8	<0.001
Physical activities	2.4 ± 4.7	0.1 ± 0.7	<0.001
Social contact	3.8 ± 6.2	0.2 ± 1.4	<0.001
Sleeping	2.8 ± 4.6	0.2 ± 0.9	<0.001
Smiling	2.3 ± 5.0	0.8 ± 2.3	0.001
Emotional status	6.4 ± 6.9	0.9 ± 2.1	<0.001
Overall	22.9 ± 18.3	4.3 ± 5.5	<0.001

^a^Student’s t test; ^b^OHIP-14: Oral Health Impact Profile; ^c^the effect size of the difference was 1.38 (95% CI: 1.12-1.64); ^d^OIDP: Oral Impacts on Daily Performances; ^e^the effect size of the difference was 1.38 (95% CI: 1.12-1.64).

There were no statistically significant differences in any outcome variables regarding to the origin of controls (data no shown).

Table 4 presents multiple regression analyses results. Patients had worse Physical Component Summary scores than controls in the unadjusted model. This relationship remained significant in all the adjusted models [β-coefficient = −0.11 (95% CI: − (−5.12)-(−0.16) in the fully adjusted model]. Conversely, the differences in Mental Component Summary between patients and controls were not statistically significant in none of the models. There were significant differences between the two groups in OHRQoL. Patients had worse OHRQoL than controls. Patients had 11.63 (95% CI: 6.77-20.01) higher odds for the OHIP-14 and 21.26 (95% CI: 11.54-39.13) higher odds for OIDP of being in a worse category (higher number of performances impaired) of OHRQoL compared to controls in the fully adjusted model. Regarding the clinical importance, the effect sizes of the differences in OHRQoL between patients and controls were 1.38 for the OHIP-14 (95% CI: 1.12-1.64) and 1.38 for the OIDP (95% CI: 1.12-1.64) (note that calculations arrived to exactly the same 95%-CIs for the OHIP and the OIDP although they come from different figures). The respective effect sizes in HRQoL were 0.28 (95% CI: 0.05-0.51) in the Physical Component Summary and 0.08 (95% CI: −0.15-0.31) in the Mental Component Summary score.

**Table 4:** **Multiple regression analysis: PCS** ^**c**^ **, MCS** ^**d**^ **, OHIP-14** ^**f**^ **and OIDP** ^**g**^ **as dependent variables**

	**1:Crude**		**2: Model 1 + Age + gender + social class**		**3: Model 2 + PFT**		**4: Model 3 + Illness**	
	**β** ^**a**^ **[95% CI]**	**p-value**	**β [95% CI]**	**p-value**	**β [95% CI]**	**p-value**	**β [95% CI]**	**p-value**
PCS^b, c^	−3.24 [−5.95-(−0.53)]	0.019	−3.87 [−6.40-(−1.33)]	0.003	−2.66 [−5.18-(−0.13)]	0.039	−2.60 [−5.10-(−0.11)]	0.041
MCS^b,d^	−0.81 [−3.22-1.60]	0.509	−0.75 [−3.18-1.69]	0.547	−0.78 [−3.28-1.72]	0.541	−0.77 [−3.27-1.74]	0.547
OHIP^e,f^	10.62 [6.30-17.90]	<0.001	11.44 [6.69-19.55]	<0.001	11.78 [6.86-20.21]	<0.001	11.63 [6.77-20.01]	<0.001
OIDP^e,g^	18.82 [10.37-34.15]	<0.001	21.07 [11.48-38.63]	<0.001	21.14 [11.52-38.82]	<0.001	21.26 [11.54-39.13]	<0.001

^a^Differences between oral cancer survivors (n = 142) and controls (n = 142); ^b^Linear regression; ^c^PCS: Physical Component Summary; ^d^MCS: Mental Component Summary; ^e^Ordinal logistic regression; ^f^OHIP: Oral Health Impact Profile; ^g^OIDP: Oral Impacts on Daily Performances.

Table 5 shows the bivariate associations between clinical and treatment data and HRQoL and OHRQoL in patients treated for oral cancer. There were statistically significant differences in the OHRQoL according to the clinical stage and type of treatment. Patients in early stages of oral cancer had better OHRQoL compared to patients with oral cancer in advanced clinical stages. Patients who received only surgical treatment obtained better scores in the OHRQoL than those that received combined treatment (surgery and radiotherapy and/or chemotherapy).

**Table 5:** **Association between clinical and treatment data and health-related quality of life and oral health-related quality of life in patients survivors of oral cancer (n = 142)**

**Variable**	**PCS** ^**a**^	**MCS** ^**b**^	**OHIP-14** ^**c**^	**OIDP** ^**d**^
	**Mean ± de**	**Mean ± de**	**Mean ± de**	**Mean ± de**
Location				
Oral	47.5 ± 11.6	36.9 ± 9.0	18.3 ± 11.9	22.1 ± 18.0
Oropharynx	46.5 ± 10.5	35.4 ± 9.0	23.7 ± 9.8	28.7 ± 20.3
	*p* ^*e*^ = 0.730	*p* ^e^ = 0.520	*p* ^g^ = 0.043	*p* ^g^ = 0.155
Clinical stage				
I-II	49.1 ± 11.9	36.5 ± 10.0	14.8 ± 10.3	17.0 ± 15.7
III-IV	46.2 ± 11.0	36.1 ± 8.1	22.0 ± 11.9	27.4 ± 19.0
	*p* ^e^ = 0.133	*p* ^e^ = 0.768	*p* ^g^ = <0.001	*p* ^g^ = <0.001
Follow-up (years)				
0.5-5	41.5 ± 1.3	46.0 ± 1.2	19.5 ± 1.3	23.4 ± 1.9
6-10	43.5 ± 2.1	44.7 ± 2.3	16.5 ± 1.7	20.1 ± 2.7
11-20	43.7 ± 2.8	47.0 ± 2.2	20.8 ± 3.2	25.6 ± 5.8
	*p* ^f^ = 0.614	*p* ^f^ = 0.780	*p* ^h^ = 0.454	*p* ^h^ = 0.743
Treatment				
Surgery	47.5 ± 12.1	36.7 ± 9.6	16.1 ± 11.6	19.0 ± 18.4
Combined treatment	47.3 ± 10.8	36.8 ± 8.2	22.0 ± 11.3	26.7 ± 17.6
	*p* ^e^ = 0.897	*p* ^e^ = 0.950	*p* ^g^ = 0.002	*p* ^g^ = 0.002

^a^PCS: Physical Component Summary; ^b^MCS: Mental Component Summary; ^c^OHIP-14: Oral Health Impact Profile; ^d^OIDP: Oral Impacts on Daily Performances; ^e^Student’s t test; ^f^ANOVA test; ^g^Mann–Whitney U test; ^h^Kruskal Wallis test.

## Discussion

This study showed that patients treated for oral or oropharyngeal cancer experienced significantly worse physical domains of HRQoL and OHRQoL compared to the general population even after adjusting for the effect of sociodemographic characteristics, oral health and general health. These differences were moderate for the physical domains of HRQoL and severe for OHRQoL. On the other hand, the results suggest that there could be a psychological adaptation over time in these patients.

Generic HRQoL measures have been widely used to compare general and patient populations, estimate the burden of disease and provide information on the effectiveness of treatments and health care [17]. The particularity of the SF-12 is that its standardized scores permit direct interpretation compared to the Spanish population norms, the reference population in this case. Thus, the patients of our sample had worse scores in all the SF-12 domains compared to the Spanish population norms [16]. This finding is similar to that found in the study of Fang et al. [8]. Comparing to the control group, we found that the mean scores of the eight functional domains and summary components of the SF were lower for patients, being statistically significant in the Role Physical, Bodily Pain, General Health in our study. These findings contrast with those found in other studies [10-13] where some domains as General Health or Vitality were better in the patients than the reference group. These disagreements could be explained because of using population norms rather than a control group or doing the evaluations at a different period after the treatment. In fact, it is difficult to know at when exactly the quality of life of patients treated for oral cancer improves [4].

The differences in Physical functioning between patients and controls were not statistically significant in our study. Maybe this could be because the items of this dimension refer to activities that require moderate efforts. There were statistically significant differences between patients and controls in the Role Physical and in the Physical component summary (even after adjusting this last variable for age, gender, social class, posterior functional teeth and illness). These differences are clinically relevant: effect sizes of the differences in SF-12 Physical component summary between patients and controls were moderate, according to the benchmarks suggested by Cohen’s standard criteria [14]. The SF-12 Physical component summary has been showed to be predictive of long-term survival in patients with head and neck cancer. A gradual increase of physical activity in these patients might have a positive impact in physical and functional domains and, in turn, the rates of overall mortality could improve [25,26].

The SF-12 Mental component summary scores are very similar in patients and controls. While it seems logical to expect that patients treated for oral cancer have considerable psychological impacts, research has shown that this is not necessarily the case [11]. Individual attitudes are modified by expectations and adaptation to the condition [27] and specifically, in oral cancer patients, they can also be influenced by coping and fear of recurrence [28,29]. Our patients were evaluated at least 6 months after treatment which could be sufficient for allowing patients time to adapt to their new situation. Moreover, patients had similar scores in Role Emotional and Social functioning to the controls in our sample; people with high scores on these domains are more likely to report better life satisfaction [11].

Despite the time elapsed since treatment and in line with the study of Hassel et al. [9], OHRQoL was significantly worse in patients than controls. The most important differences, both in OHIP-14 and OIDP, were found in items associated with eating, a finding similar to that in the study by Linsen et al. [30], and speaking. Problems eating could be directly linked to the frequent reports of difficulty chewing and swallowing in patients treated for oral cancer [4,31,32]. On the other hand, the problem with speaking could be due to restriction in tongue mobility (the most frequent location of oral cancer in our sample). It can result in speech incomprehension and is highly correlated with patient’s quality of life [33]. These differences between patients and controls should not be underestimated as the very large effects sizes (both for OHIP and OIDP) highlighted their clinical importance. The aforementioned consequences would improve if preventive rehabilitation programs were routinely established for these patients [34-36].

Looking at the clinical characteristics of the oral cancer patients, we showed that combined therapy (as opposed to only surgical treatment) and advanced (compared to early) clinical stages of oral cancer adversely affected the OHRQoL of the patients. These results agree with those found in previous studies [37-39]. There is no consensus in the literature about whether the location of the tumor affects the quality of life [39]. In our study, it was a variable significantly associated with OHRQoL evaluated with the OHIP-14; oropharyngeal cancer patients had worse OHRQoL than oral cancer patients. Finally, the follow-up period was not significantly associated with any of these parameters. This finding together with the results of other studies showing improvements in quality of life after a year [40], three years [37] or five years [10,12,13], suggest that the literature is inconclusive about a specific follow-up period associated with improved HRQOL or OHRQoL and also imply that the pattern of improvement over time may not be linear.

This study has some limitations. First, there is not relevant data available to allow comparisons with pre-treatment scores, which would have shown to what extent our results reflect the long-term adaptation of patients after treatment. We have attempted to partly address this by including a control group in order to compare estimates between this and the group that received treatment. We also used both generic HRQoL and OHRQoL questionnaires, but their use does not rule out the possibility that the observed impacts in patients may be due to other oral conditions, not just due to oral cancer or its treatment. This could have been addressed through the additional use of cancer-specific HRQoL and OHRQoL measures and these should be included in future research. Second, we also acknowledge that the heterogeneity in terms of the follow-up period for the patients treated with cancer may not be suitable for determining critical time periods for evaluation of quality of life [13]. However, this variety in the follow-up period reflects more accurately what actually happens with the population of oral cancer patients and therefore our results are more representative of the perceptions and experiences of patients surviving from oral cancer. A third limitation relates to the selection of controls. We did not undertake a random selection of the base population but this is acceptable practice when the base population is difficult to identify. In addition, we employed methodological features to account for potential limitations linked to selection of controls. We matched for sex and age in order to minimize confounding and further tested this in the bivariate analysis. Moreover, there were no differences in the outcomes with respect to the origin of controls. The information was collected in the same way in cases and controls (comparable accuracy) and we recruited a relatively large sample with the same number of controls and cases which allowed us to achieve the objectives of the study (efficiency).

## Conclusion

This study indicated that oral cancer patients had worse OHRQoL and worse scores in physical dimensions of HRQoL than the general population at least 6 months after treatment. Conversely, they were similar to the general population in overall psychological dimensions of HRQoL, possibly due to psychological adaptation to their condition. Implemention of rehabilitation programs could benefit and improve the quality of life of these patients.

## Abbreviations

HRQoL: Health-related quality of life
OHRQoL: Oral health-related quality of life
SF-12: The 12-item short form health survey
OHIP-14: Oral health impact profile
OIDP: Oral impacts on daily performances
